# Better Together: Interorganellar Communication in the Regulation of Proteostasis

**DOI:** 10.1177/25152564241272245

**Published:** 2024-10-08

**Authors:** Andreas Kohler, Verena Kohler

**Affiliations:** 1 Department of Medical Biochemistry and Biophysics, 8075 Umeå University, 901 87 Umeå, Sweden; 2 Department of Molecular Biology, 8075 Umeå University, 901 87 Umeå, Sweden

**Keywords:** protein homeostasis, reactive oxygen species, stress response, chaperones, protein misfolding, calcium

## Abstract

An extensive network of chaperones and folding factors is responsible for maintaining a functional proteome, which is the basis for cellular life. The underlying proteostatic mechanisms are not isolated within organelles, rather they are connected over organellar borders via signalling processes or direct association via contact sites. This review aims to provide a conceptual understanding of proteostatic mechanisms across organelle borders, not focussing on individual organelles. This discussion highlights the precision of these finely tuned systems, emphasising the complicated balance between cellular protection and adaptation to stress. In this review, we discuss widely accepted aspects while shedding light on newly discovered perspectives.

## Introduction

Cellular proteostasis is a delicate balance responsible for protein synthesis, maintenance and degradation. Various pathways are instrumental in regulating this equilibrium, from the machinery required for protein synthesis to the systems responsible for breaking down misfolded or unused proteins (Hipp et al., 2019). While protein quality control factors are present in every cellular compartment, these individual systems must be functionally interconnected and coordinated to uphold proteostasis. Interorganellar communication, a critical aspect of cellular function, describes the dynamic interplay between organelles, an interaction facilitated through physical associations via contact sites but also through signalling mechanisms (reviewed in (Prinz et al., 2020; Carreras-Sureda et al., 2022; Jain and Zoncu, 2022)). Accurate sorting between cytosolic proteins and proteins destined for organelles, like mitochondria or the endoplasmic reticulum (ER), followed by translocation across organellar boundaries, plays essential roles in guaranteeing optimal function. The mitochondrial proteome, with its dual genetic origin, requires precise coordination of cytosolic and mitochondrial protein production in addition to the tight control of nuclear-encoded mitochondrial protein import (reviewed in (Lee-Glover and Shutt, 2023; den Brave et al., 2024)). The ER is a major site for processing and folding proteins, including integral membrane proteins and proteins destined for the secretory pathway. Protein import into the ER occurs through conserved co- and post-translational mechanisms (reviewed in (Aviram and Schuldiner, 2017)), characterized by the involvement of important proteostasis factors. Maintaining proteostasis necessitates a close association with energy conversion. ATP, the universal energy source essential for life and almost all cellular functions, is for example used to support protein folding, but also for phosphorylation, trafficking and degradation in the ER (Dorner et al., 1990; Braakman et al., 1992; Dorner and Kaufman, 1994; Quéméneur et al., 1994; Hirschberg et al., 1998; Rosser et al., 2004). ATP transport from mitochondria to the ER relies on a sarcoplasmic/endoplasmic reticulum Ca-ATPase (SERCA)-dependent calcium gradient across the ER membrane, increasing mitochondrial ATP regeneration while decreasing glycolysis (Wescott et al., 2019; Yong et al., 2019; Boyman et al., 2020). Calcium microdomains are postulated to form at ER-mitochondria contact sites, stimulating mitochondrial respiration to encourage efficient ATP production, especially under ER stress when protein misfolding increases ATP uptake from mitochondria (Bravo et al., 2011; Kaufman and Malhotra, 2014; Yong et al., 2019). Elevated cytosolic calcium levels are proposed to impede ATP import into the ER lumen (Yong et al., 2019). Understanding the organisation of the cellular proteostasis network and its responsiveness to both exogenous and endogenous stressors holds fundamental significance in biology and medicine (proteostasis mechanisms reviewed in (Kohler and Andréasson, 2023)).

The objective of this review is to elucidate the communication between organelles, whether through signalling processes such as the integrated stress response (ISR), unfolded protein response (UPR) and the heat shock response (HSR) or via direct physical contact sites and to illuminate their role in maintaining proteostasis. We provide an overview of established communication pathways, highlight open research questions and explore the emerging role of direct organellar contacts.

### The Integrated Stress Response – a Global Stress Sensing Mechanism

**Figure 1.**
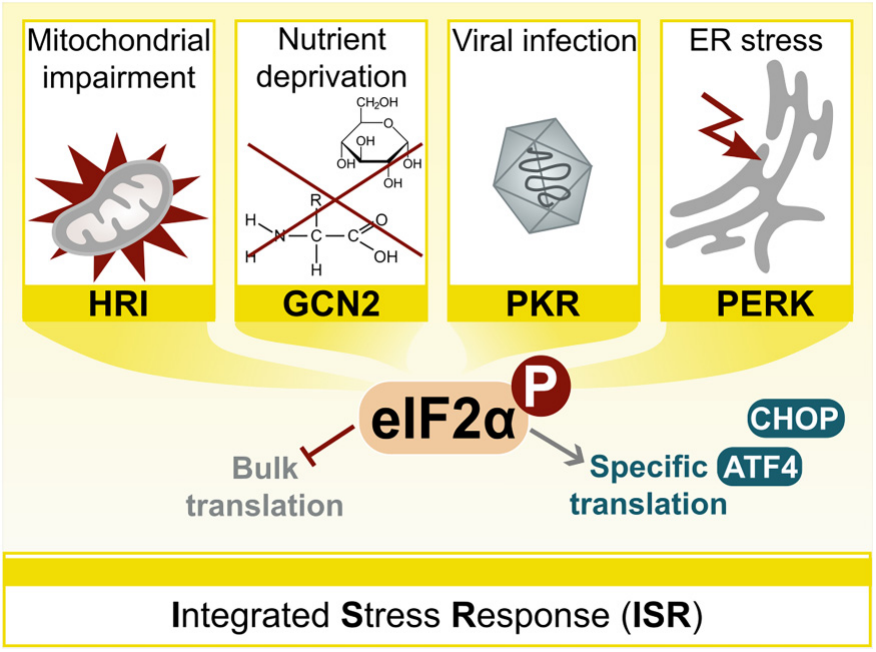
Scheme of the integrated stress response (ISR). The ISR responds to a wide range of changes by activating 4 different stress-specific kinases: HRI, GCN2, PKR and PERK, leading to phosphorylation of the eukaryotic initiation factor 2α (eIF2α). Please see the main text for further details. ER = endoplasmic reticulum.

A prominent example of interorganellar communication responding to potentially detrimental, proteotoxic stressors is the ISR (Costa-Mattioli and Walter, 2020). It presents a conserved signalling network over several organellar borders that responds to physiological and pathological changes, encompassing external factors like hypoxia leading to mitochondrial dysfunction (via heme-regulated inhibitor; HRI), amino acid or glucose deprivation (via general control nonderepressible 2; GCN2), viral infections (via protein kinase R; PKR), as well as intrinsic cues like ER stress (via PKR-like endoplasmic reticulum kinase; PERK) (Figure 1) (Balachandran et al., 2000; Dong et al., 2000; Harding, Zhang et al., 2000; Han et al., 2001; Pakos-Zebrucka et al., 2016; Costa-Mattioli and Walter, 2020; Kaspar et al., 2021). The regulatory domains of the mentioned factors respond to their respective stress signals by activating their kinase domains via dimerization and trans-autophosphorylation. This leads to the phosphorylation of the eukaryotic initiation factor 2α (eIF2α), a critical event causing reduced global protein synthesis, while translating key genes for cellular recovery, including the activating transcription factor 4 (ATF4), a well-described ISR effector, as well as the C/EBP homologous protein (CHOP), a transcription factor determining cellular fates (Figure 1) (Palam et al., 2011; Fiorese et al., 2016; Pakos-Zebrucka et al., 2016; Quirós et al., 2017; Liu et al., 2024). The ISR is also described in the context of mitochondrial defects, underscoring its widespread importance (Tyynismaa et al., 2010; Dogan et al., 2014; Bao et al., 2016; Nikkanen et al., 2016). Recently, the potential for pharmacological activations of compensatory eIF2α kinases to rescue ISR signalling and promote mitochondrial adaption has been suggested (Perea, Baron et al., 2023). While the UPR identifies misfolded proteins within the ER and the HSR is triggered by cytosolic protein aggregation, the ISR is activated by stress signals originating from both the ER and the cytosol. Consequently, the initiation of the ISR can be intricately linked to the activation of both stress responses (Costa-Mattioli and Walter, 2020). While components of the ISR play a role at organellar contacts, further studies are required to assess a potential direct involvement of contact sites.

### The Unfolded Protein Response in the Endoplasmic reticulum

**Figure 2.**
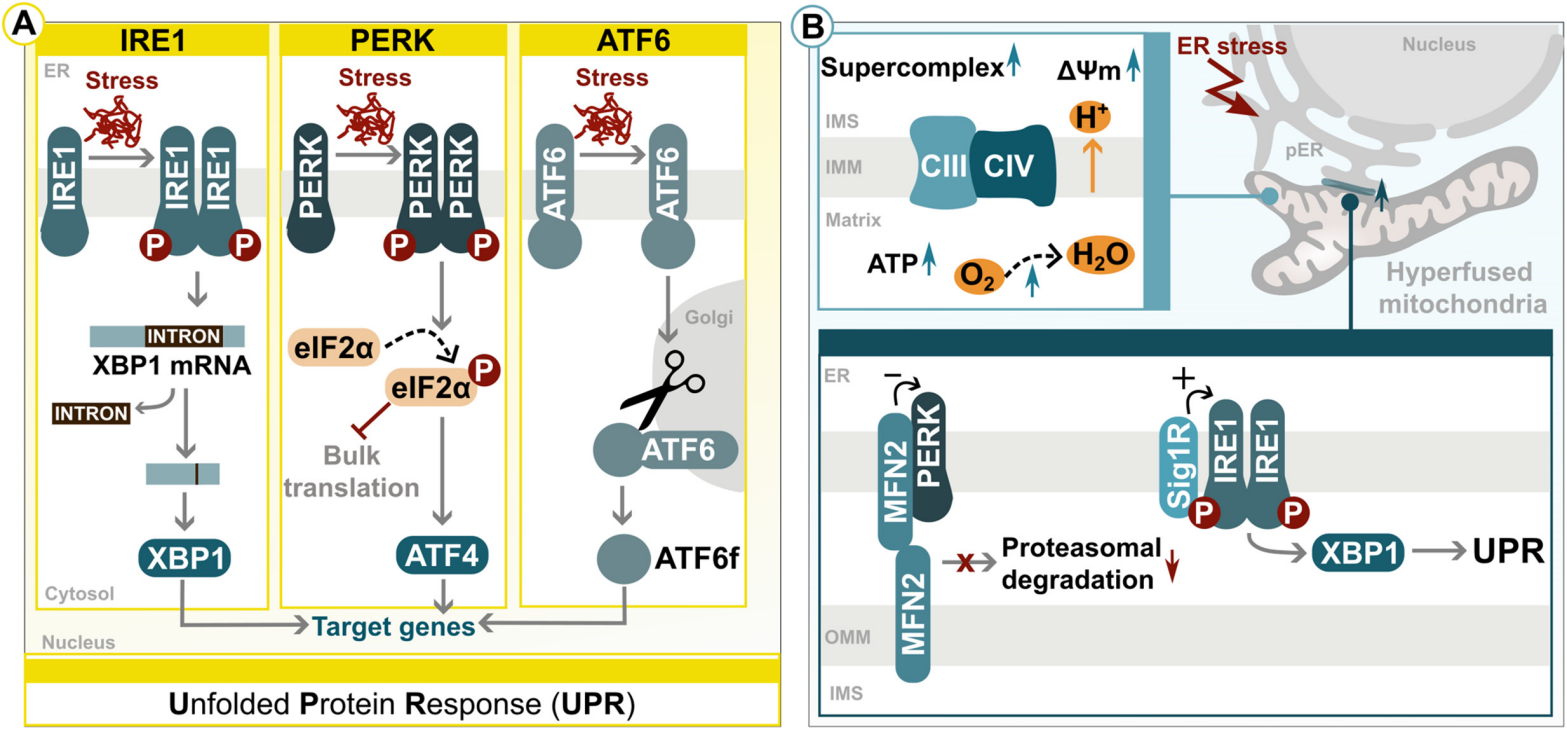
Mechanisms of the unfolded protein response (UPR). (A) Protein misfolding stress leads to activation of the UPR via signalling cascades from three main effectors: IRE1, PERK and ATF6. (B) During ER stress, contacts between mitochondria and ER increase, stimulating mitochondrial respiratory activity and the UPR via interplay between ER-resident chaperones and UPR sensors. Please see main text for further details. P = phosphorylation; eIF2α = eukaryotic initiation factor 2α; pER = perinuclear endoplasmic reticulum; IMS = intermembrane space; IMM = inner mitochondrial membrane; OMM = outer mitochondrial membrane; ΔΨm = mitochondrial transmembrane potential; CIII = respiratory complex III; CIV = respiratory complex IV.

In response to ER stress, cells activate the UPR, a transcriptional pathway conveying signals from the ER to the nucleus. It operates in two phases: first reducing protein synthesis and enhancing the degradation of misfolded proteins; then, initiating transcriptional upregulation, involving hundreds of target genes responsible for controlling global proteostasis (Hetz et al., 2020). The UPR was initially discovered in yeast, centred around the inositol-requiring enzyme 1 (IRE1) sensor. In mammals, there are three distinct branches of UPR activation, executed by IRE1, PERK and the activating transcription factor 6 (ATF6), all resulting in the upregulation of specific target genes, including those coding for ER-associated degradation (ERAD) and folding factors (reviewed in (Hwang and Qi, 2018). Both IRE1 and PERK undergo dimerization and trans-autophosphorylation upon detection of misfolded proteins. For IRE1, this results in the activation of its RNase domain, resulting in splicing and production of the transcription factor X-box binding protein 1 (XBP1; (Figure 2A)) (Cox and Walter, 1996; Yoshida et al., 2001; Calfon et al., 2002; Korennykh et al., 2009; Gardner and Walter, 2011). PERK conformational change inhibits general protein translation by phosphorylating eIF2α as described for ISR signalling, reducing the influx of unfolded proteins into the ER and enhancing the expression of transcription factors, including ATF4 (Figure 2A) (Harding, Novoa et al., 2000; Harding et al., 2003; Verfaillie et al., 2012). ATF6 follows a different activation routine by translocating from the ER to the Golgi apparatus, where it undergoes cleavage, liberating its cytosolic domain (ATF6f) that enters the nucleus (Figure 2A) (Shen and Prywes, 2004; Nadanaka et al., 2007; Yamamoto et al., 2007). Initially, all branches work to enhance pro-survival signalling, but if ER stress persists, this mechanism can shift towards a cell death program (Yoshida et al., 2001; Calfon et al., 2002; Bashir et al., 2021). During such an extended UPR activation, ER calcium is released at ER-mitochondria contact sites, resulting in mitochondrial transmembrane potential loss, fragmentation and apoptosis (Scorrano et al., 2003; Deniaud et al., 2008).

While the fundamental mechanism of these ER stress sensors is well characterised, increasing evidence shows that they play a significant role in facilitating and regulating communication routines and transfer processes between organelles, including e.g., calcium transfer, protein folding activities, ATP generation, as well as overall organelle homeostasis. IRE1 and PERK are suggested to support mitochondrial function and metabolism in non-stressed, basal conditions and are described to localize at ER-mitochondria contact sites (Chami et al., 2008; Bouman et al., 2011; Verfaillie et al., 2012; Tomohisa Mori et al., 2013; Carreras-Sureda et al., 2019). Early ER stress leads to mitochondrial hyperfusion and repositioning of ER and mitochondria towards the perinuclear region, enhancing their coupling and increasing mitochondrial respiratory chain supercomplex formation, oxidative phosphorylation (OXPHOS) activity and ATP production as well as calcium transfer via an active PERK axis (Figure 2B) (Csordás et al., 2006; Bravo et al., 2011; Fujimoto et al., 2012; Lebeau et al., 2018; Balsa et al., 2019; Kohler et al., 2023). Contact side-resident proteins were shown to modify UPR responses, such as the ER chaperone sigma-1 receptor (Sig1R) that transiently associates with IRE1, enhancing its RNase activity, thus promoting XBP1 mRNA splicing (Figure 2B) (Tomohisa Mori et al., 2013; Alam et al., 2017). ER stress leads to increased abundance of ER-mitochondria contacts, which can be explained by reduced proteasomal degradation of mitofusin 2 (MFN2), a key factor in tethering ER and mitochondria (Bravo et al., 2011; Ngoh et al., 2012; Chen and Dorn, 2013; Gottschalk et al., 2022; Naón et al., 2023). Moreover, MFN2 is characterized as an upstream regulator that directly interacts with PERK. This interaction negatively influences PERK activity, which in turn affects both apoptosis and autophagy during ER stress conditions (Figure 2B). While MFN2 deficiency is described to result in mitochondrial dysfunction through prolonged PERK activation, PERK silencing is shown to counteract MFN2-induced apoptosis, reducing the production of reactive oxygen species (ROS) and normalising calcium levels (Sebastián et al., 2012; Muñoz et al., 2013; Schneeberger et al., 2013).

### The Mitochondrial Unfolded Protein Response

**Figure 3.**
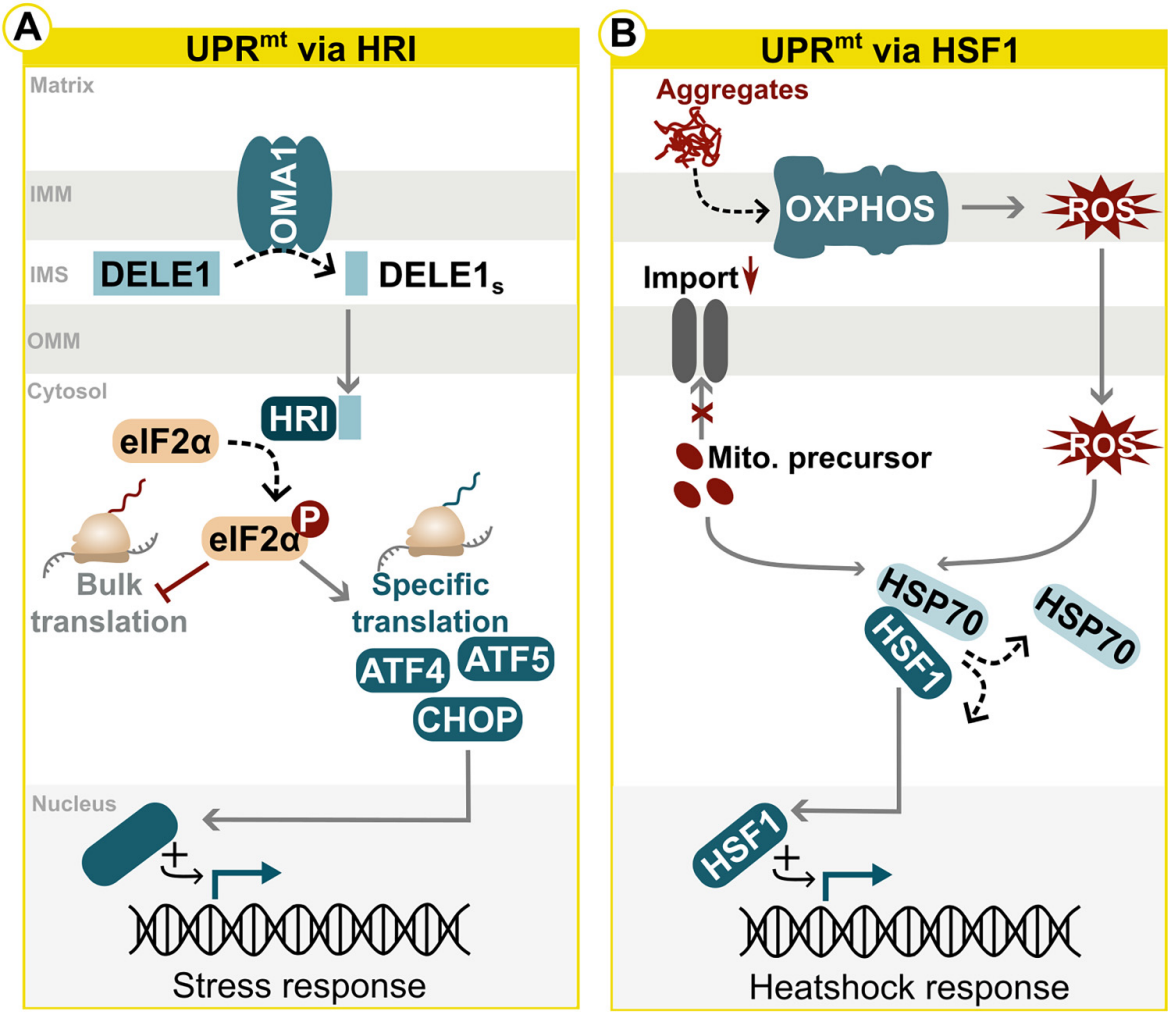
Stress response pathways in mitochondria and ER. (A, B) Mechanisms for the activation of the mitochondrial unfolded protein response (UPR^mt^) are depicted. (A) Classical activation of UPR^mt^ activation in mammals upon mitochondrial dysfunction as part of the integrated stress response (ISR). (B) Alternative activation of UPR^mt^ via HSF1 as recently demonstrated by Sutandy et al. IMS = intermembrane space; IMM = inner mitochondrial membrane; OMM = outer mitochondrial membrane; P = phosphorylation; eIF2α = eukaryotic initiation factor 2α; ROS = reactive oxygen species; Mito. precursor = mitochondrial precursor proteins.

The mitochondrial unfolded protein response (UPR^mt^), a transcriptional response to mitochondrial dysfunction, is part of the ISR in mammals with HRI as the effector kinase (Figure 1) (Fiorese et al., 2016; Münch and Harper, 2016; Quirós et al., 2017; Samluk et al., 2019). It coordinates gene expression in this dual-genome system, promoting survival and metabolic adaptation while restoring mitochondrial function. However, prolonged activation can lead to the propagation of malfunctioning mitochondrial genomes, contributing to age-related diseases, which emphasizes the need for fine-tuned activation (Zhu et al., 2021). Mitochondrial dysfunction in mammals activates the inner mitochondrial membrane-based protease OMA1, which cleaves the intermembrane space protein DELE1 into DELE1S. This fragment is exported into the cytosol, causing eIF2α phosphorylation via HRI and subsequent reduced translation of most proteins, while enabling specific translation of ATF4, ATF5 and CHOP (Figure 3A) (Fessler et al., 2020; Guo et al., 2020; Liu et al., 2024). However, recent research reveals alternative UPR^mt^ activation during mitochondrial folding stress, utilizing the HSR via the transcription factor HSF1, rather than the primary ISR effector ATF4. This activation requires a combination of signals: mitochondrial ROS release due to OXPHOS malfunction and the cytosolic accumulation of mitochondrial protein precursors due to impaired mitochondrial protein import. These signals collectively trigger UPR^mt^ by freeing HSF1 from its complex with HSP70, which then translocates to the nucleus, where it initiates the HSR (Figure 3B) (Sutandy et al., 2023). While the elements involved in signal detection and facilitation may vary across organisms (for instance, ATFS1 in *C. elegans* (Nargund et al., 2012; Rolland et al., 2019)), the outcome consistently involves mitochondria-to-nucleus communication, leading to the transcriptional upregulation of stress-related genes (Figure 3A, B). UPR^mt^ activation is not confined to the presence of unfolded proteins in mitochondria but also observed in situations such as impaired mitochondrial import, OXPHOS deficiency, stoichiometric imbalance, lipid biogenesis impairments, increased oxidative stress, ATP depletion or mitochondrial ribosome inhibition (Yoneda et al., 2004; Durieux et al., 2011; Baker et al., 2012; Nargund et al., 2012; Houtkooper et al., 2013; Moullan et al., 2015; Kim et al., 2016). However, pinpointing the exact molecular triggers for UPR^mt^ induction is complex, as mitochondrial dysfunctions often result in secondary effects that can be mistakenly identified as activators, requiring further research to dissect these molecular principles.

### The Tight Interplay Between Lipid Composition and ER Stress Response

Re-establishing ER-mitochondria contacts simultaneously with altering ER membrane fluidity disrupts IRE1 oligomerization, which attenuates its UPR response (Sanchez-Alvarez et al., 2017). *Vice versa*, UPR sensors can influence cellular lipid metabolism, extending beyond the traditional view of UPR-induced ER-membrane expansion (Moncan et al., 2021). Instead, for example changes in lipid composition, like a shift in the phosphatidylcholine: phosphatidylethanolamine ratio or an increase in saturated fatty acids coupled with a decrease in monounsaturated fatty acids in phospholipids, lead to the activation of a broad stress response via the so-called lipid bilayer stress, further detailed in (Ariyama et al., 2010; Gao et al., 2015; Fun and Thibault, 2020; Celik et al., 2023). IRE1 and PERK respond to these lipid bilayer perturbations through signals transduced via conformational changes in their ER-spanning transmembrane domain, without requiring the unfolded protein stress-sensing domain (Pineau et al., 2009; Promlek et al., 2011; Volmer et al., 2013; Tam et al., 2018; Micoogullari et al., 2020; Chattopadhyay et al., 2021; Kennelly et al., 2021). MFN2, a main player involved in ER-mitochondria contacts and the UPR, was additionally shown to specifically bind and extract phosphatidylserine by isolating it from membranes and forming rigid domains enriched in this lipid to promote its transfer to mitochondria, where phosphatidylethanolamine synthesis occurs, likely via favouring the activity of phosphatidylserine transport proteins (Maeda et al., 2013). Thus, this protein plays an important role in proper lipid metabolism and ER homeostasis (Hernández-Alvarez et al., 2019). The UPR sensor PERK also promotes lipid shuttling at ER-mitochondria contact sites in a UPR-independent manner, operating as a recruiter for Extended Synaptogamin 1 (E-Syt1), a protein facilitating the calcium-dependent transfer of glycerolipids between membrane bilayers (Saheki et al., 2016). Compromising this interaction leads to decreased mitochondrial respiration and impaired lipid transfer, thus PERK plays an essential role in maintaining mitochondrial health (Sassano et al., 2023). PERK, along with its downstream targets eIF2α, ATF4 and CHOP, is further described to promote the adaptive remodelling of the mitochondrial membrane by accumulating phosphatidic acid on the outer mitochondrial membrane, thus inhibiting fission and inducing protective mitochondrial elongation during ER stress (Perea, Cole et al., 2023). Lipid bilayer stress is known to affect ER membranes, leading to the destabilization of transmembrane proteins, which are then degraded via ERAD (Shyu et al., 2019). One such target is SERCA which is inactivated upon lipid perturbations e.g., via increasing levels of phosphatidylcholine which results in disrupted calcium homeostasis, leading to ER calcium depletion and triggering chronic ER stress. This mechanism is initially independent of the luminal stress-sensing domains of the UPR sensors (Li et al., 2004; Fu et al., 2011). However, this situation interferes with calcium-dependent chaperones and enzymes for protein folding, subsequently activating the UPR via conventional pathways (Promlek et al., 2011; Volmer et al., 2013).

### Interorganellar Calcium Signalling in Cellular Stress Response

**Figure 4.**
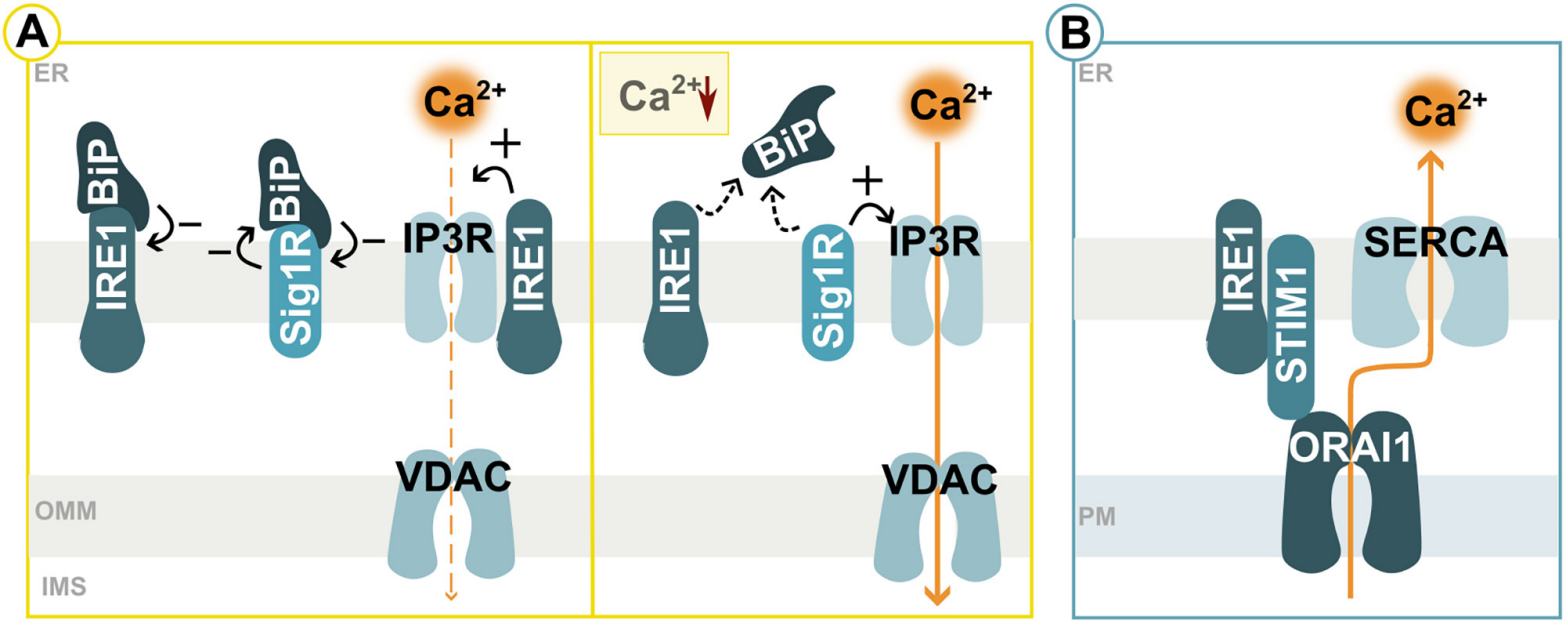
Cellular response to ER calcium levels. (A) ER calcium levels determine the binding strengths of the ER-resident chaperone BiP to its partners and the activity of the calcium channel IP3R via fine-tuning by IRE1 and Sig1R. (B) A simplified mechanism of store-opened calcium entry (SOCE) as a response to low ER calcium level is depicted. Please see main text for more details. IMS = intermembrane space; OMM = outer mitochondrial membrane; PM = plasma membrane.

Calcium homeostasis is vital for cellular processes, with multiple organelles involved to maintain physiological levels, including ER, mitochondria and lysosomes, and enable quick modifications for signalling. ER-mitochondria contacts are key to sustaining basal calcium levels, being essential for UPR regulation and overall proteostasis functionality. Components of the ER protein folding machinery have low-affinity calcium binding sites, reducing calcium occupancy upon low ER calcium levels, triggering the activation of UPR, with several possibly redundant models described. Under physiological conditions, Binding Immunoglobulin Protein (BiP), an ER-resident HSP70 chaperone (Kohler and Andréasson, 2020), represses ER stress sensors via binding their luminal domains (Bertolotti et al., 2000; Amin-Wetzel et al., 2017, 2019). During ER stress, BiP disengages with these UPR sensors, facilitating their activation (Figure 4A) (Bertolotti et al., 2000; Hetz et al., 2020). The ER transmembrane protein El24 aligns with the IRE1 branch of the UPR through similar processes. In the absence of stress, it binds IRE1, preventing its activation, but detaches upon stress occurrence to target the calcium channel inositol trisphosphate receptor (IP3R) to avert ER calcium depletion (Xu et al., 2022). The release of ER calcium is associated with the disruption of specific BiP-substrate complexes, further promoting UPR sensor activation (Preissler et al., 2017, 2020). Calcium increases the affinity of BiP for ADP, hence, in a calcium-abundant environment, substrate retention is favoured, while depleted conditions lead to efficient substrate-BiP disengagement. This process is suggested to link ER quality control to signals facilitating ER calcium mobilisation (Preissler et al., 2020). Similarly, Sig1R and BiP form a complex under normal conditions, which keeps both factors inactive. Low ER calcium levels induce rapid disassembly of Sig1-BiP, increasing chaperone activity. Sig1R interacts with and stabilises IP3R, increasing calcium flux towards mitochondria, supporting ATP synthesis, thus forming a calcium-sensitive chaperone system (Figure 4A) (Szabadkai et al., 2006; Hayashi and Su, 2007; Mavlyutov et al., 2010). In this line, Sig1R facilitates ER-mitochondrial calcium transfer and mitigates ER stress when overexpressed, while its loss induces ER-stress-mediated cell death (Hayashi and Su, 2007; Wu and Bowen, 2008; Happy et al., 2015; Morihara et al., 2018). Here, Sig1R additionally coordinates the coupling of ankyrin B to control calcium signalling (Hayashi and Su, 2007). IRE1 also influences the extent of calcium transfer by serving as a scaffold for IP3R at ER-mitochondria contact sites. IP3R acts as a tether by contacting Sig1R and VDAC1 (Atakpa-Adaji and Ivanova, 2023), implying that IRE1 might indirectly promote the formation of ER-mitochondria contact sites by docking to IP3R, thereby enhancing calcium transfer essential for mitochondrial respiration and ATP production (Figure 4A) (Bartok et al., 2019; Carreras-Sureda et al., 2019). IP3R degradation is partially governed by ER luminal calcium, representing another layer of regulation (Bhanumathy et al., 2006).

ER calcium depletion triggers the formation of higher-order Stromal interaction molecule 1 (STIM1) oligomers, a conserved ER-resident calcium sensor, leading to its redistribution to the cortical ER, where it induces oligomerization of the calcium-release activated calcium channel protein, the plasma-membrane-resident ORAI1, highlighting the role of ER-plasma membrane contacts in these processes (Roos et al., 2005; Zhang et al., 2005). Additionally, in a dynamic trapping mechanism, STIM1 binding to the microtubule plus end-binding protein EB1 that restricts STIM1 to ER-PM junctions (Chang et al., 2018). This happens simultaneously with a significant ER architecture reorganization. ORAI1 channel activation initiates the influx of extracellular calcium through Store-operated Calcium Entry (SOCE), a ubiquitous mechanism for calcium influx in mammalian cells (reviewed in (Lopez et al., 2020)). Calcium is then pumped into the ER via SERCA, ending SOCE and ensuring an adequate calcium microenvironment (Figure 4B) (Orci et al., 2009). IRE1 interacts with STIM1 to promote ER-plasma membrane contact assembly, STIM-ORAI1 interactions and SOCE, linking these mechanisms (Carreras-Sureda et al., 2023). Similarly, PERK also plays a UPR-independent role in response to calcium levels, regulating ER-plasma membrane contact sites (van Vliet et al., 2017). Increased cytosolic calcium levels trigger PERK dimerization without UPR, leading to its interaction with filamin A to control the F-actin network under the plasma membrane. This coordination repositions STIM1 and E-Syt1, facilitating efficient ER-plasma membrane contacts and subsequent SOCE (van Vliet et al., 2017). E-Syt1 plays a role in calcium-dependent lipid transfer at ER-plasma membrane contacts, tethers peroxisomes to the ER, facilitating cholesterol transport via lysosome-peroxisome-ER contacts (Xiao et al., 2019). E-Syt1 is further enriched at ER-mitochondria contact sites, where its deletion reduces the contact site number and length but also leads to impaired calcium flux and an altered mitochondrial lipidome (Giordano et al., 2013; Xiao et al., 2019; Ge et al., 2022; Janer et al., 2024).

ER-lysosome contacts act as central hubs, enabling calcium mobilisation and signalling events, which play a major role in central proteostasis mechanisms, including protein folding and UPR. The second messenger nicotinic acid adenine dinucleotide phosphate (NAADP) orchestrates the organised release of calcium, serving as an IP3R agonist that initiates calcium release from the ER (Churchill and Galione, 2001; Kinnear et al., 2004; Zong et al., 2009; Penny et al., 2014). Establishing ER-lysosome contacts and maintaining optimal lysosome pH are suggested to be crucial in supporting bidirectional calcium signalling control, a key aspect of cellular processes, (Kilpatrick et al., 2013; Morgan et al., 2013; Atakpa et al., 2018). In situations of mitochondrial calcium overload, a regulated cell death mechanism is triggered via the activation of the mitochondrial permeability pore. A pro-survival mechanism involves the upregulation of the stress-induced ROS-generating protein NOX4 at ER-mitochondria contact sites. This inhibits IP3R-mediated calcium transfer via phosphorylation, creating spatially delimited redox signalling at these contact sites that prevents cell death (Beretta et al., 2020).

### The Interplay Between Redox Mechanisms, Calcium Signalling and Stress Response

ER and mitochondria are important intracellular redox hubs, facilitating redox-related communication. The ER with oxidative folding may be the most significant cellular ROS producer, with mitochondrial OXPHOS also contributing significantly to its generation (Murphy, 2008; Malinouski et al., 2011; Hudson et al., 2015). Interestingly, ER oxidative protein folding regulates mitochondrial metabolism and mitochondrially-produced ROS increases ER stress, reinforcing ROS production in both organelles, emphasizing this tight interplay (Simmen et al., 2010; Yoon et al., 2011). Single mitochondria generate oxidative bursts at ER-mitochondria contact sites, which are sensed by calcium channels like IP3R, triggering calcium transients. Stressors or apoptotic stimuli increase the frequency and amplitude of these bursts (Booth et al., 2021). Redox regulatory proteins (chaperones and oxidoreductases) like calnexin, ER oxidoreductin 1 (ERO1) and selenoprotein N1 (SEPN1) are specifically enriched at ER-mitochondria contacts (Gilady et al., 2010; Yoboue et al., 2017). Some produce ROS themselves, facilitating essential interorganellar signal transmission processes and binding to calcium-handling proteins in a redox-dependent manner, regulating calcium flux and subsequently mitochondrial metabolism (Myhill et al., 2008; Li et al., 2009; Anelli et al., 2012; Lynes et al., 2013; Marino et al., 2015; Gutiérrez and Simmen, 2018). IP3R, SERCA as well as mitochondrial calcium uniporters are redox-sensitive, thus ROS levels directly influence calcium flux. ROS at ER-mitochondria contacts lead to SERCA oxidation and inactivation, while activating IP3R, resulting in a feed-forward loop for ER-mitochondria calcium flux (Qin et al., 1998; Li et al., 2011; Raturi et al., 2014). The oxidoreductase ERdj5 and SEPN1 counteract SERCA oxidation, thus activating this channel, while Thioredoxin Related Transmembrane Protein 1 (TMX1) inactivates SERCA and reduces cytosolic calcium uptake (Marino et al., 2015; Raturi et al., 2016; Ushioda et al., 2016). IP3R is described to be regulated by two potentially competing proteins, ERp44, interacting with IP3R under resting conditions to inhibit ER calcium release, and ERO1, a protein leading to disulfide bond generation in newly synthesized proteins, which increases ER calcium release (Tu and Weissman, 2002; Higo et al., 2005; Li et al., 2009; Anelli et al., 2012). ERO1 was also shown to interact with PERK during early ER stress. This complex increases the contacts between ER and mitochondria, maintains mitochondrial calcium bioenergetics and promotes the oxidation of proteins at ER-mitochondrial contact sites, thus improving ER-mitochondria calcium flux and limiting oxidative stress (Bassot et al., 2023). IRE1 is also strategically positioned at these contact sites, as ROS activate the IRE1-XBP1 branch without affecting ATF6 and PERK (Tomohisa Mori et al., 2013), again highlighting the interconnection and the specificity of proteostasis responses.

### Cellular Response to Mislocalized Proteins

**Figure 5.**
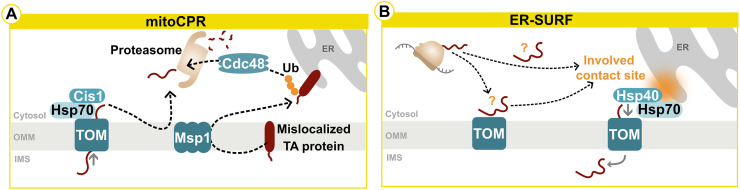
Interorganellar communication upon mitochondrial protein import failures and general proteotoxic stress. (A) The mitochondrial compromised import response (mitoCPR) pathway is an important mechanism upon impairment of the import of nuclear-encoded mitochondrial proteins. (B) The ER surface-mediated targeting (ER-SURF) pathway is involved in targeting hydrophobic precursor proteins to the ER surface and ER-mitochondria contacts facilitate the relocalization to mitochondria. It is not entirely clear if targeted proteins failed to be imported into mitochondria or if this route is used directly for an initial attempt to import proteins into mitochondria. Please see the main text for further details. IMS = intermembrane space; OMM = outer mitochondrial membrane; Ub = ubiquitination; TA protein = tail-anchored protein.

The accumulation of non-imported precursor proteins in the cytosol due to mitochondrial dysfunction can challenge homeostasis, thus components of different surveillance mechanisms span over several cellular compartments. The mitochondrial TOM complex, essential for mitochondrial protein import, is constantly monitored, e.g., by Ubx2 in yeast (Schuberth and Buchberger, 2005). Ubx2 is a component of mitochondrial protein translocation-associated degradation (mitoTAD), where it binds to the TOM complex, recruiting the ATPases Associated with diverse cellular Activities (AAA-ATPase) Cdc48 in the cytosol to extract proteins that block the translocase for subsequent degradation (Mårtensson et al., 2019). This process operates alongside the mitochondria-localized ribosome-associated quality control (mitoRQC), which handles obstructed precursor proteins co-translationally inserted and associated with stalled ribosomes (Izawa et al., 2017; Su et al., 2019). While extensively characterized in yeast, similar mechanisms are expected in higher eukaryotes due to the conservation of fundamental proteostasis processes. In line with other protein quality control mechanisms, a certain redundancy with backup mechanisms is also observed in this case, again best characterized in yeast. Artificial induction of mitochondrial import deficiency by introducing a clogging protein leads to significant reconfiguration of the proteostatic system (Boos et al., 2019). This response, orchestrated by Hsf1, upregulates various chaperones, including the key proteasome regulator Rpn4, enhancing proteasome assembly and activity. This mechanism is an upstream regulator for two pathways: mitochondrial compromised import response (mitoCPR) and unfolded protein response activated by the mistargeting of proteins (UPR^am^) (Wrobel et al., 2015; Weidberg and Amon, 2018). In mitoCPR, Cis1 expression is induced, leading to its association with the TOM complex. The AAA-ATPase Msp1, a direct interactor of Cis1, collaborates with the proteasome to eliminate mislocalized precursor proteins (Figure 5A) (Weidberg and Amon, 2018). Notably, Msp1 can extract misdirected tail-anchored proteins from the outer mitochondrial membrane, facilitating their transport to the ER involving the Guided Entry of Tail-anchored (GET) ER import pathway, where their proteasomal degradation is initiated via Cdc48 (Matsumoto et al., 2019, 2022). Similarly, the proteasome gets activated by the UPR^am^ upon increased levels of mitochondrial precursor proteins in the cytosol (Wrobel et al., 2015). The authors describe a proteasome activation proportional to the quantity of mislocalized precursors, providing a cytoprotective buffering system for mitochondria, again underlining the importance of cross-compartmental surveillance for proteostasis.

When mitochondrial import is deficient, the ER surface-mediated targeting (ER-SURF) pathway directs hydrophobic precursor proteins to the ER surface, maintaining their transport competence before transfer to mitochondria, specifically to the TOM receptors Tom22 and Tom70, by chaperones of the Hsp40 family, Djp1 and Xdj1 (Figure 5B) (Hansen et al., 2018; Opaliński et al., 2018). This suggests that the ER-SURF may serve as a quality control mechanism for re-importing previously non-imported mitochondrial proteins (Lahiri et al., 2014; Elbaz-Alon et al., 2015; Lang et al., 2015; Kreimendahl and Rassow, 2020; Koch et al., 2021). Recent evidence shows a direct involvement of ER-mitochondria contact sites in facilitating the targeting of mitochondrial precursor proteins from the ER to mitochondria via two parallel pathways. One is via the yeast ER-mitochondria encounter structure (ERMES) and the other via Tom70, together with the contact site regulator Lam6 and the Hsp40 Djp1. Here, hydrophobic inner membrane proteins particularly rely on this mechanism. ERMES and Djp1 are proposed to not only provide proximity but also positively influence the biogenesis or stability of mitochondrial matrix and inner membrane proteins. However, the potential impact of defective mitochondrial lipid composition in contact site mutants cannot be excluded at this time (Koch et al., 2024). Tom70 on the mitochondrial surface is suggested to be more of a recruiter of cytosolic chaperones, reducing the proteotoxicity of hydrophobic mitochondrial membrane proteins, rather than an import receptor (Backes et al., 2021), with further research needed to unravel the molecular mechanisms. Distinguishing between intentional ER surface localization for initial mitochondrial targeting and mislocalization due to import defects can be challenging, as non-imported mitochondrial proteins accumulate in various cellular compartments, including the ER but also the nucleus (Shakya et al., 2021; Knöringer et al., 2023). While mitochondrial membrane proteins are routed to the ER under physiological conditions, this phenomenon is enhanced by import defects and stimuli increasing protein expression. The ER seems to be a physiological buffer zone for mitochondrial precursors not fit for immediate import with the UPR as an important tool to adjust ER proteostasis. Mislocalized proteins additionally induce the ISR, substantiating the global effect of mitochondrial dysfunction (Bao et al., 2016; Quirós et al., 2017; Forsström et al., 2019). Distressed mitochondria communicate with peroxisomes through upregulated Acyl CoA Oxidase 2 (ACOX2), a newly identified peroxisomal ISR marker regulated by ATF4. This interplay illuminates the interconnected responses of organelles and systemic reactions, seen via a crosstalk between the nucleus, ER and peroxisomes (Neupane et al., 2022). In this line, impairment of peroxisomes induces ER stress, ERAD and increases eIF2α phosphorylation (Kovacs et al., 2009, 2012; Cai et al., 2018). As neither XBP1 splicing nor IRE1 signalling is involved, this points towards the activation of a specific branch of UPR, most likely the PERK axis, as this transducer is part of the ISR (Kovacs et al., 2009, 2012; Huang et al., 2019). Even though evidence is pointing towards an important role of peroxisomes during stress responses and in intracellular signalling, we are just beginning to explore the underlying mechanisms.

In response to accumulation of non-imported proteins, an alternative strategy involves spatial sequestration of misfolded, proteotoxic protein species into potentially inert storage places. Sequestration compartments have been described in both yeast and mammalian cells, and several involved chaperones and pathways seem to be conserved (Rolli and Sontag, 2022). Here, the involvement of interorganellar communication in these proteostasis processes is just beginning to be explored. Q-bodies, a dynamic quality control compartment in the yeast cytosol, colocalize with Vps13, an important facilitator of contact sites (Escusa-Toret et al., 2013; Eisele et al., 2021). A recent study shows that the reversible juxtanuclear quality control compartment (JUNQ) and intranuclear quality control compartment (INQ) are localized at contact sites between the nucleus and the vacuole, the yeast counterpart of lysosomes, and are surrounded by mitochondria, suggesting coordination between the proteostasis machinery and organelles (Sontag et al., 2023). Interestingly, a recent study introduces MitoStores as cytosolic protein granules storing non-imported mitochondrial precursors (Krämer et al., 2023). Even though clearly distinguished from other sequestration compartments harbouring amyloids, these quality control compartments share several similarities with JUNQ, including their transient nature and the presence of certain proteostasis factors. Further research is needed to explore potential parallels or distinctions between MitoStores and JUNQ and to confirm the potential role of mitochondria-ER contact sites in managing proteostasis for mitochondrial proteins, especially regarding the handling of non-imported mitochondrial polypeptides.

*Vice versa*, mitochondria seem to be an important sequestration space for both cytosolic and ER-resident proteins. Misfolded proteins formed in the yeast ER were shown to be transported to, sequestered and finally degraded within mitochondria via a pathway termed ER-associated mitochondrial sequestration (ERAMS), however with the impairment of mitochondrial function coming at a considerable cost. This pathway is suggested to function via mitochondria-ER contact sites, as prominent factors in protein import are not involved (Cortés Sanchón et al., 2021). A similar concept was described in the handling of aggregation-prone cytosolic proteins in yeast that enter mitochondria for degradation. This mitochondria-mediated proteostasis mechanism taking care of cytosolic cargo was termed mitochondria as guardian in cytosol (MAGIC) and some evidence shows that it might also exist in human cells (Ruan et al., 2017). Both concepts seem to favour degradation over refolding after using a whole organelle as the sequestration host, thus it will be interesting to explore if ERAMS and MAGIC function as a last resort after failure of other proteostasis concepts.

### Interorganellar Communication and Degradative Capacity

Protein degradation is vital for proteostasis, with lysosomes capable of breaking down various cargo, transported by macroautophagy, chaperone-mediated autophagy and endocytosis (Kohler and Andréasson, 2023). Autophagy, an evolutionarily conserved process, plays a crucial role in maintaining protein balance, degrading misfolded proteins and cooperating with the proteasome in cellular proteostasis (Walker and Ktistakis, 2020; Yim and Mizushima, 2020; Aman et al., 2021; Kaushik et al., 2021). Subsequently, lysosomal enzymatic hydrolysis yields essential amino acids that are recycled for other cellular processes (Jackson and Hewitt, 2016; Yim and Mizushima, 2020). Interorganellar communication emerges as a decisive orchestrator of autophagy regulation, specifically for the formation of the phagophore and the involved channelling of membrane resources as specified in a recent review (Gómez-Sánchez et al., 2021). This encompasses physical interactions, including ER-autolysosome, ER-mitochondria, ER-plasma membrane, ER-phagophore-lysosome, and nucleus-vacuole contacts (details extensively reviewed in (Kohler et al., 2020; Duckney et al., 2022; Zwilling and Reggiori, 2022; Capitanio et al., 2023; Enrich et al., 2023)).

### Outlook and Open Questions

Our review explores the complex interplay between interorganellar communication and proteostasis, highlighting their multifaceted roles in cellular functions. While most research insights come from studies on the ER and mitochondria it is essential to recognise that additional organelle combinations likely also play roles in stress management and proteostasis. The idea of direct organellar contacts contributing to these processes holds intriguing potential. We recognize the significant role of lipid metabolism and the general composition of lipid membranes in cellular stress response, with lipid bilayer stress as a recognized branch activating the UPR. However, the role of lipid droplets in the context of proteostasis is just beginning to be explored. Lipid droplets have been observed to clear misfolded proteins from both the cytosol and the ER (Moldavski et al., 2015; Vevea et al., 2015; Henne et al., 2018), while cells lacking lipid droplets showed a reduced efficiency in eliminating misfolded proteins (Moldavski et al., 2015). Although evidence suggests their involvement in stress responses and influencing autophagy regulation (Puskás et al., 2010; Bosma et al., 2014; Velázquez et al., 2016; Chen et al., 2017; Chitraju et al., 2017; Mukhopadhyay et al., 2017; Jarc and Petan, 2019), it remains to be clearly shown whether these processes are direct or indirect, e.g., by engulfing potentially highly hydrophobic proteins within lipid droplets or by alleviating stress caused by lipid imbalances and potentially toxic lipid species that strain the general stress response system. However, the molecular details and signalling cascades in this context remain elusive. It will be an exciting future task to characterize the molecular fundamentals to include these compartments into the regime of stress-involved organelles like the ER, mitochondria, peroxisomes and lysosomes.

The concept of interorganellar communication and the role of contact sites in coordinating a cell-wide stress response are essential but come with trade-offs. Particularly in protein misfolding-related diseases like neurodegenerative disorders, isolation of affected organelles might be a protective mechanism via the downregulation of major signalling routes. Research findings highlight the delicate balance at play, with some controversies based on the system under investigation and expression levels. Our review has primarily focused on individual contact sites in cellular pathways. However, we propose the existence of multiple concurrent contact sites and interorganellar communication systems. Just as mitochondria-vacuole contacts serve as backup routes for ER-mitochondria contacts in yeast (Elbaz-Alon et al., 2014; Mao et al., 2022), it is plausible that other organellar contact sites and communication systems act as backup or complementary pathways. Developing a comprehensive understanding of these backup mechanisms can reveal additional layers of the complex network of interorganellar communication, offering exciting opportunities for further research and discovery.

## Abbreviations

AAA-ATPase: ATPases Associated with diverse cellular Activities
ACOX2: Acyl CoA Oxidase 2
ATF4: Activating transcription factor 4
ATF6: Activating transcription factor 6
BiP: Binding Immunoglobulin Protein
CIII: Respiratory complex III
CIV: Respiratory complex IV
cER: Cortical Endoplasmic Reticulum
CHOP: C/EBP homologous protein
eIF2α: Eukaryotic initiation factor 2 α
ER: Endoplasmic Reticulum
ERAD: Endoplasmic Reticulum-Associated Protein Degradation
ERMES: ER-mitochondria encounter structure
ERO1: ER oxidoreductin 1
ER-SURF: ER Surface-mediated Targeting
E-Syt1: Extended Synaptogamin 1
GCN2: General control nonderepressible 2
GET: Guided entry of tail-anchored proteins
HRI: Heme-regulated inhibitor
HSR: Heat shock response
IMS: intermembrane space
IMM: inner mitochondrial membrane
INQ: Intranuclear Quality Control Compartments
IPOD: Insoluble Protein Deposits
IP3R: Inositol trisphosphate receptor
IRE1: Inositol-requiring enzyme 1
ISR: Integrated Stress Response
JUNQ: Juxtanuclear Quality Control Compartments
mitoCPR: Mitochondrial Compromised Import Response
MFN2: Mitofusin 2
MitoRQC: Mitochondria-localized ribosome-associated quality control
mitoTAD: Mitochondrial Translocation-Associated Degradation
MPP: Mitochondrial Pre-Sequence Protease
NAC: Nascent polypeptide-associated complex
NAADP: Nicotinic acid adenine dinucleotide phosphate
OMM: Outer mitochondrial membrane
OXPHOS: Oxidative phosphorylation
pER: Perinuclear endoplasmic reticulum
PERK: PKR-like endoplasmic reticulum kinase
PKR: Protein kinase R
PMCA: Plasma membrane Ca^2+^ ATPase
Q-bodies: Quinary bodies
ROS: Reactive Oxygen Species
SEPN1: Selenoprotein N1
SERCA: Sarcoplasmic/endoplasmic reticulum Ca^2+^-ATPase
Sig1R: Sigma-1 receptor
SRP: Signal Recognition Particle
STIM1: Stromal interaction molecule 1
TMX1: Thioredoxin Related Transmembrane Protein 1
TOM: Translocase of the Outer Membrane
UPR: Unfolded Protein Response
UPR^am^: Unfolded Protein Response Activated by Mistargeting of Proteins
UPR^mt^: Mitochondrial Unfolded Protein Response
XBP1: X-box binding protein 1
ΔΨm: Mitochondrial transmembrane potential
